# Monte Carlo microdosimetry of ^211^At in multi-species thyroid follicle models: AlfaMC versus MCNPX and Geant4

**DOI:** 10.1186/s40658-026-00925-w

**Published:** 2026-08-15

**Authors:** Arman Romiani, Anders Josefsson, Roumiana Chakarova, Eva Forssell-Aronsson

**Affiliations:** 1 Department of Medical Radiation Sciences, Institute of Clinical Sciences, Sahlgrenska Academy at University of Gothenburg, 413 45 Gothenburg, Sweden; 2 Department of Biomedical Engineering and Medical Physics, Sahlgrenska University Hospital, 413 45 Gothenburg, Sweden; 3 Sahlgrenska Center for Cancer Research, Sahlgrenska Academy at University of Gothenburg, 405 30 Gothenburg, Sweden

**Keywords:** Alpha particles, AlfaMC, MCNPX, Geant4, Thyroid follicle, Microdosimetry

## Abstract

**Background:**

Radionuclide therapy is an increasingly used treatment modality for patients with disseminated cancer diseases. Tumor-binding radiopharmaceuticals labelled with the alpha-particle emitter astatine-211 (^211^At) are of interest to enable high radiation absorbed doses to small tumors. The alpha particles emitted by ^211^At will deposit all their energy in a short-range including neighboring cells, causing localized irradiation. Due to being part of group 17 the halogen family with iodine, free ^211^At accumulates in the thyroid gland, which is the main normal organ at risk. It is therefore of great importance to estimate the radiation absorbed dose accurately to the thyroid gland for free ^211^At.

The aims of the work were to determine microdosimetric quantities for uniform and non-uniform 211At distributions within different models of the thyroid gland using the Monte Carlo (MC) codes AlfaMC and GEometry ANd Tracking (Geant4), and to compare the results with previously published data using Monte Carlo N-Particle eXtended (MCNPX). AlfaMC is customized for calculating microdosimetric quantities for alpha-particles.

**Results:**

Both a single and a multiple thyroid follicle model designed for man, rat and mouse were used from previously published studies. Monte Carlo simulations and absorbed dose-calculations were performed for different distributions of 211At within the thyroid models. The mean specific energy, single-hit mean specific energy and single-hit specific energy distribution were calculated for follicle cell nuclei in models for each species.

**Conclusion:**

The overall agreement between AlfaMC and MCNPX results, and between AlfaMC and Geant4 results, was found to be within 5% and 2%, respectively. The intercomparison suggested an effective way for validation of microdosimetry results; of AlfaMC validation, a code designed with a focus on reducing the calculation time.

## Introduction

Radionuclide therapy, using radionuclides either in free form (usually as ions) or attached to tumor targeting molecules, is appropriate for treatment of patients with metastatic tumor diseases. The requirement of such therapy is that the absorbed dose in tumor tissue is high enough compared with that in normal organs and tissues.

Astatine-211 (^211^At) is an alpha-particle emitter well suited for radionuclide therapy with a half-life of 7.2 h emitting one net alpha-particle through two possible decay pathways to stable lead-207 (^207^Pb). X-rays with energies up to 92 keV are also emitted during decay, allowing external measurement of ^211^At [1]. The two main energies of the emitted alpha-particles are 5.867 MeV (yield 41.8%) and 7.450 MeV (yield 58.2%). Due to the short-range of the emitted alpha-particles (up to 70 μm in water) and high linear energy transfer (LET) (60–230 keV/µm) [2], the frequency of DNA double strand breaks (DSB) and more complex DNA damage are higher than that caused by beta-particle emitters (e.g. ^177^Lu), most probably resulting in higher cell death frequency. Furthermore, the short-range of the emitted alpha-particles results in very low absorbed dose to adjacent normal tissues. These properties make ^211^At interesting for cancer treatment and especially for small tumors [3].

^211^At has been proposed and tested clinically for cancer treatment since the 1950s, in most cases bound to various tumor-seeking agents and in some cases as free ^211^At. Clinical studies have been performed or are in progress or in the planning stage [4, 5]. Many ^211^At-labelled agents have been developed and tested preclinically, e.g., alpha-melanocyte stimulating hormone peptide analog against melanoma and several vectors against glioblastoma and prostate cancer [6–9].

In vivo, ^211^At-labelled compounds may undergo deastatination and ”free ^211^At” will be circulating in the body, to a high degree as astatide, but most probably also in other valence states. Studies on mice and rats have shown that free ^211^At is largely accumulated in the thyroid gland, but the biodistribution differs from that of radioiodide [10, 11]. In man, relatively high uptake of free ^211^At was found in patients with thyroid disorders as early as 1954 [12]. The thyroid gland is therefore considered to be one of the normal organs with the highest risk for side effects in radionuclide therapy involving ^211^At labelled agents.

The short-range of alpha-particles in combination with high LET results in a large variation in energy deposition in different neighboring cells. Therefore, it may be inaccurate to use the mean absorbed dose to the tumor or organs at risk when estimating biological effects from the radiation. Microdosimetry considers the stochastic nature of the energy deposited and is therefore more appropriate for alpha-particle irradiation.

There are different methods for calculating microdosimetric quantities. It is possible to develop a simplified simulation of alpha-particle transport assuming continuous slowing down in a straight trajectory and neglecting energy- and angular straggling. The energy difference of the alpha-particle, before and after traversing e.g. a cell, is assumed to represent the deposited energy in that cell. Such calculations can be made on a spreadsheet and do not require a certain Monte Carlo (MC) program [13].

More sophisticated MC techniques use random numbers to simulate the stochastic process of the radiation transport and apply probability density functions to describe the outcome of each interaction process. Different MC codes are available that can simulate alpha-particle transport. The general-purpose code Monte Carlo N-Particle eXtended (MCNPX), developed at the Los Alamos National Laboratory [14], and the object-oriented MC toolkit GEometry And Tracking (Geant4) developed at the Conseil Européen pour la Recherche Nucléaire (CERN) [15] have been widely used [16, 17]. The Geant4-DNA project extended the Geant4 toolkit to calculations at molecular level useful in radiobiology and radiation therapy [18].

Both these MC codes are capable to simulate the transport of more than 30 different particles in complex geometry. However, code installation, correct choice of physical processes and design of input files reflecting the task objective, require special expertise and programming skills. MCNPX defines the task in terms of input cards. Geant4 requires good knowledge of C + + object-oriented computer language and control. Whereas Geant4 is an open-source code, the MCNP distribution is restricted. Also, the speed of the general-purpose codes may not be optimal for a particular problem addressed by a specialized code like AlfaMC [19].

AlfaMC is a MC code dedicated only to alpha-particles and was introduced by Luis Peralta and Alina Louro at the Science faculty of the University of Lisbon. The code was originally developed for irradiation of cells and customized for calculation of microdosimetric quantities. It uses Continuous Slowing Down Approximation (CSDA) and considers energy and angular straggling. Powerful geometrical software package is included, based on combinatorial geometry. A great advantage over other more complex MC codes is the short calculation time. Stopping power calculations, identified as the most frequent operations in the code, were optimized by applying precomputed lookup Table [19]. A typical simulation run tracking 100,000 alpha particles took about 2 min to complete [20]. AlfaMC has shown good agreement with the SRIM code for estimation of energy deposition in thin layers for alpha-particles with kinetic energies between 1 MeV and 12 MeV [19].

Thyroid models for man, rat and mouse were previously developed and applied for dosimetric analysis of ^211^At [21, 22] and radioiodine as ^131^I, ^125^I, ^124^I, and ^123^I [22, 23]. In those studies, MCNPX was used to investigate how different factors such as the distribution of the radiohalogens and the geometrical dimensions of respective models affect microdosimetric quantities. Since experimental validation of the results is not possible, an inter-comparison of independent theoretical data is of importance.

The aim of this work was to determine microdosimetric quantities for uniform and non-uniform ^211^At distributions within different thyroid follicle models by using the MC codes AlfaMC and Geant4 and to compare the results with previously published MCNPX data [21] for the same models.

## Methods

Previously published single and multiple thyroid follicle models for man, rat and mouse [21] were defined in AlfaMC and Geant4 codes using the corresponding geometry tools.

### Thyroid follicle models

#### Single follicle model

**Fig. 1:**
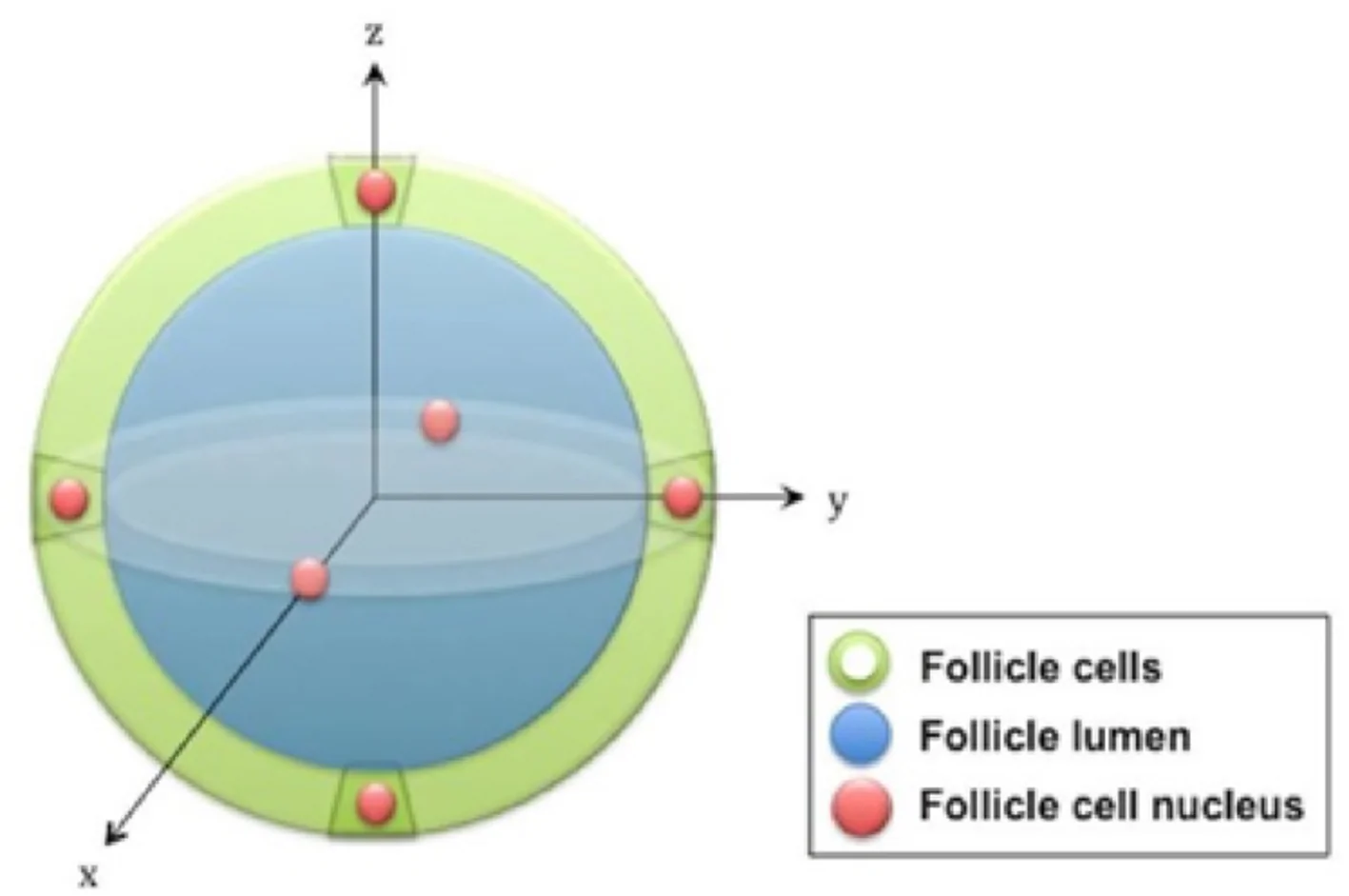
Schematic figure of the single thyroid follicle model [21]. The follicle cells, the follicle lumen and the follicle cell nuclei are shown in green, blue and red, respectively

The single thyroid follicle models for man, rat and mouse consisted of a single sphere-shaped thyroid follicle with a spherical follicle lumen, a layer of follicle cells (shaped as a shell around the follicle lumen) and six spherical follicle cell nuclei distributed symmetrically at equal distances from the center (Fig. 1) [21]. Each component had liquid water density (1.0 g/cm^3^). The diameter of the follicle lumen, thickness of the follicle cell layer and the diameter of the follicle cell nucleus were 150, 10, 8 μm; 70, 8, 6 μm; and 50, 6, 4 μm for man, rat and mouse, respectively.

#### Multiple follicle model

**Fig. 2:**
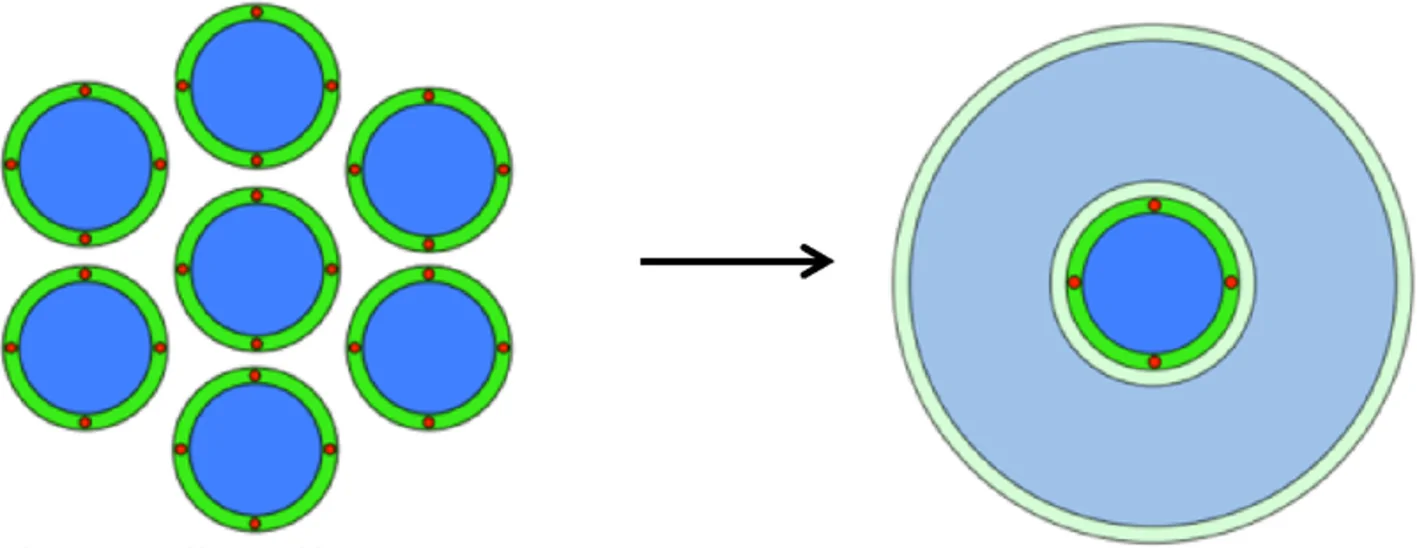
Schematic figure of seven thyroid follicles (left) and their simplification in the multiple thyroid follicle model (right) [21]. The follicle cells, the follicle lumen and the follicle cell nuclei are shown in green, blue and red, respectively. Surrounding follicle lumen and surrounding cell are shown in light blue and light green, respectively

The previously published multiple thyroid follicle model for respective species represented a simplification of the case where the thyroid follicle is surrounded by neighboring follicles (Fig. 2-left). The follicle cells from the neighboring cells formed both a spherical shell around the follicle cells in the center follicle and around their merged lumens (Fig. 2-right). The surrounding follicle lumen and surrounding cell in Fig. 2b had a thickness of 150 and 10 μm for man, 70 and 8 μm for the rat, and 50 and 6 μm for the mouse, respectively.

### Sources

Only the two main energies for the emitted alpha-particle, 5.867 MeV (yield 41.8%) and 7.450 MeV (yield 58.2%) were considered in this work. Isotropic emission of alpha-particles was assumed. Four different ^211^At distributions were used for simulations: (1) ^211^At uniformly distributed within the follicle lumen, (2) ^211^At uniformly distributed within the follicle cells, (3) ^211^At uniformly distributed within the follicle cell nuclei and (4) ^211^At uniformly distributed on the surface of concentric spheres, with their center located at the center of the follicle lumen. The radius of the spheres varied from 0 to 150 μm with 5 μm increments, hence spheres were both within the follicle lumen and in some cases outside the layer of follicle cells. This simulation represented heterogeneous distribution of ^211^At.

These four source localizations were possible for free astatine, where localizations no. 1, 2 and 4 were the most likely ones [24]. When astatine was bound to a pharmaceutical, the situation was best reflected by localization no. 4 (concentric spheres representing the basolateral membrane of the follicle cells).

In all simulations the targets were the six follicle cell nuclei.

### Monte Carlo simulations

Several microdosimetric parameters were determined as presented below. More detailed description of the quantities can be found in the International Commission on Radiation Units (ICRU) report 36 [25]. MC simulations were performed using AlfaMC and Geant4 and the results were compared to previously published data using MCNPX [21, 22].

#### AlfaMC

The Monte Carlo code AlfaMC was used for all simulation cases [19]. AlfaMC was installed on Ubuntu 14.04 LTS, with processor Intel^®^ Core™ i7-4770 and CPU @ 3.40 GHz × 8, OS type 64-bit. The code was based on the CSDA in consideration of energy- and angular straggling and used the stopping-power values from National Institute of Standards and Technology (NIST) ASTAR database [26]. The energy straggling function selected for each simulation was based on a Gaussian/Vavilov/Landau model. For each calculation 10^7^ alpha-particles were followed, and the cut-off energy was set to 1 keV. Six independent microdosimetric estimates for the six targets were obtained per one simulation. By repeating the simulation twice, twelve independent values were determined and statistically analyzed.

The thyroid follicle models were defined by combining basic volumes available in the geometric package of AlfaMC. The organization of the volumes used a mother-daughter system, which allowed the user to create volumes inside each other. For example, the follicle cell nuclei were “daughters” of the follicle cells in Fig. 1. To ensure that the geometry of the model was correct a tool component, SHOWTRACK, was used. The source distribution was also examined using SHOWTRACK (not shown).

The scoring in AlfaMC was programmed so that the energy imparted, expressed in MeV, in each follicle cell nucleus was multiplied by a conversion factor 1.602ˑ10^− 13^ (J/MeV) and divided by the mass, m, (kg), of the nucleus yielding the specific energy,

1 $$ \frac{ \epsilon }{m} \cdot 1.602 \cdot 10^{{ - 13}} = z,\left[ {Gy} \right]. $$

The sum of the specific energy in each target was either divided by the total number of simulated alpha-particles $\:\left(N\right)$ to calculate the mean specificenergy,

2 $$ \left\langle z \right\rangle = \frac{1}{N}\sum {_{{i = 1}}^{N} } z_{i} ,\left[ {Gy} \right], $$

or divided by the number of alpha-particles that have hit the target $\:\left(M\right)$ to calculate the single-hit mean specific energy,

3 $$ \left\langle {z_{1} } \right\rangle = \frac{1}{M}\sum {_{{i = 1}}^{M} } z_{i} ,\left[ {Gy} \right]. $$

One simulation returned six output values of $\left\langle z \right\rangle$ and $\left\langle {z_{1} } \right\rangle$ expressed in Gy, related to the six follicle cell nuclei targets. Each simulation, except the case where ^211^At was uniformly distributed on the surface of concentric spheres, was repeated twice with different random seeds to retain twelve output values, hence increasing the statistical accuracy. The output values were used to calculate a mean standard deviation for respective parameters.

The frequency histograms in AlfaMC were used for describing the single-hit mean specific energy distribution. The distribution was obtained by scoring the number of events per bin, the bin size was 20 mGy in all histograms.

#### Geant4

Geant4 was implemented in the case of single thyroid follicle model for uniform distribution of the source in the follicle lumen, cell and the six nuclei with the six nuclei as targets. Alpha-particle transport in Geant4 was simulated by using the standard physics constructor recommended for low-energy transport. Test calculations were performed for lumen as a source by activating discrete physical interactions in the target region; nuclear scattering by the G4DNAIonElasticModel, excitation by the DNAMillerGreenExcitationModel, ionization by the DNARuddIonisationModel and electron capture by the DNADingfelderChargeDecreaseModel. Standard low energy physics was applied elsewhere in the simulation geometry [15, 16, 18].

The geometry was defined in the DetectorConstruction-class based on the volume-in-volume concept with the largest volume called the *world volume*. Each volume was defined by G4Sphere shape with dimension corresponding to the follicle model of man, rat or mouse, at positions defined by vectors and with G4-water material. The energy deposition, given in [Gy], was collected event-by-event during the transport of each source alpha-particle and summed over the whole run. The output listed the total number of source particles in the run, (typically 5· 10^7^ particles), the absorbed dose in the scoring volume and the number of hits. The mean specific energy and the single-hit mean specific energy were calculated according to Eqs. (2) and (3).

## Results

### Single thyroid follicle models

Table 1 shows the mean specific energy, $\left\langle z \right\rangle$and the single-hit mean specific energy, $\left\langle {z_{1} } \right\rangle$calculated by AlfaMC and compared with MCNPX [21, 22] and the standard physics Geant4 for the single thyroid model for man, rat and mouse assuming different distributions of ^211^At; uniformly distributed within the nucleus, the follicle cells or the lumen. The values of $\left\langle z \right\rangle$ and $\left\langle {z_{1} } \right\rangle$ were equal in the case of ^211^At uniformly distributed within the nucleus, since the source and target were the same. The intercomparison was evaluated as the percentage difference between the results obtained by the MC codes used. The largest difference was 4.6%, which was between the rat and the human model of AlfaMC and MCNPX. The standard deviation was within 0.4% for all MC results.

**Table 1:** Mean specific energy, $\left\langle z \right\rangle$, and single-hit mean specific energy, $\left\langle {z_{1} } \right\rangle$, for single thyroid follicle models for the six nuclei as target and six nuclei as source (A); follicle cells as source (B); lumen as source (C)

(A) Nucleus → Nucleus			
	AlfaMC	MCNPX	Geant4
	$\langle z\rangle$ and $\langle {z}_{1}\rangle$ [Gy]	$\langle z\rangle$ and $\langle {z}_{1}\rangle$ [Gy]	$\langle z\rangle$ and $\langle {z}_{1}\rangle$ [Gy]
Mouse	5.20E–1	5.36E–1	5.21E–1
Rat	2.32E–1	2.40E–1	2.31E–1
Man	1.30E–1	1.36E–1	1.31E–1

	AlfaMC-MCNPX	AlfaMC-Geant4	Geant4-MCNPX
	$\langle z\rangle$ and $\langle {z}_{1}\rangle$ [%]	$\langle z\rangle$ and $\langle {z}_{1}\rangle$ [%]	$\langle z\rangle$ and $\langle {z}_{1}\rangle$ [%]
Mouse	− 3.0	− 0.2	− 2.9
Rat	− 3.3	0.4	− 3.8
Man	− 4.4	− 0.8	− 3.8

(B) Follicle cell → Nucleus						
	AlfaMC		MCNPX		Geant4	
	$\langle z\rangle$[Gy]	$\langle {z}_{1}\rangle$[Gy]	$\langle z\rangle$[Gy]	$\langle {z}_{1}\rangle$[Gy]	$\langle z\rangle$[Gy]	$\langle {z}_{1}\rangle$[Gy]
Mouse	2.63E–3	9.71E–1	2.74E–3	1.01E0	2.62E–3	9.72E–1
Rat	1.36E–3	4.43E–1	1.40E–3	4.60E–1	1.35E–3	4.42E–1
Man	3.07E–4	2.48E–1	3.17E–4	2.57E–1	3.05E–4	2.47E–1

	AlfaMC-MCNPX		AlfaMC-Geant4		Geant4-MCNPX	
	$\langle z\rangle$[%]	$\langle {z}_{1}\rangle$[%]	$\langle z\rangle$[%]	$\langle {z}_{1}\rangle$[%]	$\langle z\rangle$[%]	$\langle {z}_{1}\rangle$[%]
Mouse	− 4.0	− 3.9	0.4	− 0.1	− 4.6	− 3.9
Rat	− 2.9	− 3.7	0.7	0.2	− 3.7	− 4.1
Man	− 3.2	− 3.5	0.6	0.4	− 3.8	− 4.0

(C) Lumen → Nucleus						
	AlfaMC		MCNPX		Geant4	
	$\langle z\rangle$[Gy]	$\langle {z}_{1}\rangle$[Gy]	$\langle z\rangle$[Gy]	$\langle {z}_{1}\rangle$[Gy]	$\langle z\rangle$[Gy]	$\langle {z}_{1}\rangle$[Gy]
Mouse	1.95E–3	1.20E0	2.03E–3	1.25E0	1.94E–3	1.20E0
Rat	1.04E–3	5.86E–1	1.08E–3	6.14E–1	1.05E–3	5.90E–1
Man	1.68E–4	3.53E–1	1.71E–4	3.70E–1	1.69E–4	3.59E–1

	AlfaMC-MCNPX		AlfaMC-Geant4		Geant4-MCNPX	
	$\langle z\rangle$[%]	$\langle {z}_{1}\rangle$[%]	$\langle z\rangle$[%]	$\langle {z}_{1}\rangle$[%]	$\langle z\rangle$[%]	$\langle {z}_{1}\rangle$[%]
Mouse	− 3.9	− 4.0	0.5	0.0	− 4.4	− 4.0
Rat	− 3.7	− 4.6	− 1.0	− 0.7	− 2.8	3.9
Man	− 1.8	− 4.6	− 0.6	− 1.7	− 1.2	− 3.0

The differences between Geant4 results using the standard low energy physics and these applying DNA physics in the target region in the case of lumen as a source were within the statistical uncertainty of 1%.

**Fig. 3:**
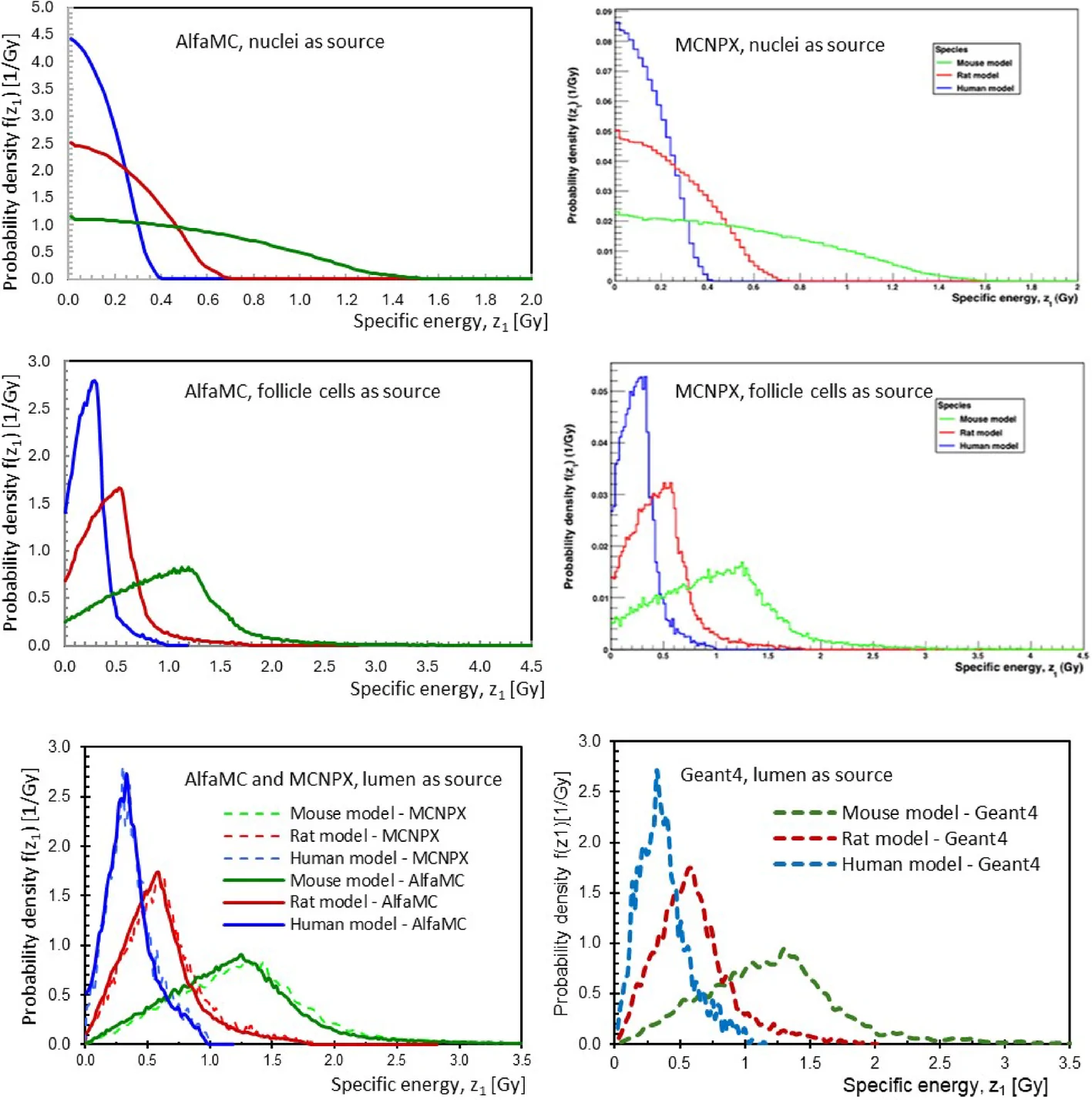
Single-hit mean specific energy distribution in single thyroid follicle models of mouse, rat and man with the nuclei, follicle cells and lumen as source obtained by AlfaMC and MCNPX [21, 22] and Geant4. Due to different normalizations the scales of y-axes differ

The single-hit specific energy distribution for the three source distributions and respective species model obtained by AlfaMC agreed with that determined by MCNPX [21, 22] and Geant4 (Fig. 3). The probability distributions were normalized so that the area under the curves became equal to 1.

**Fig. 4:**
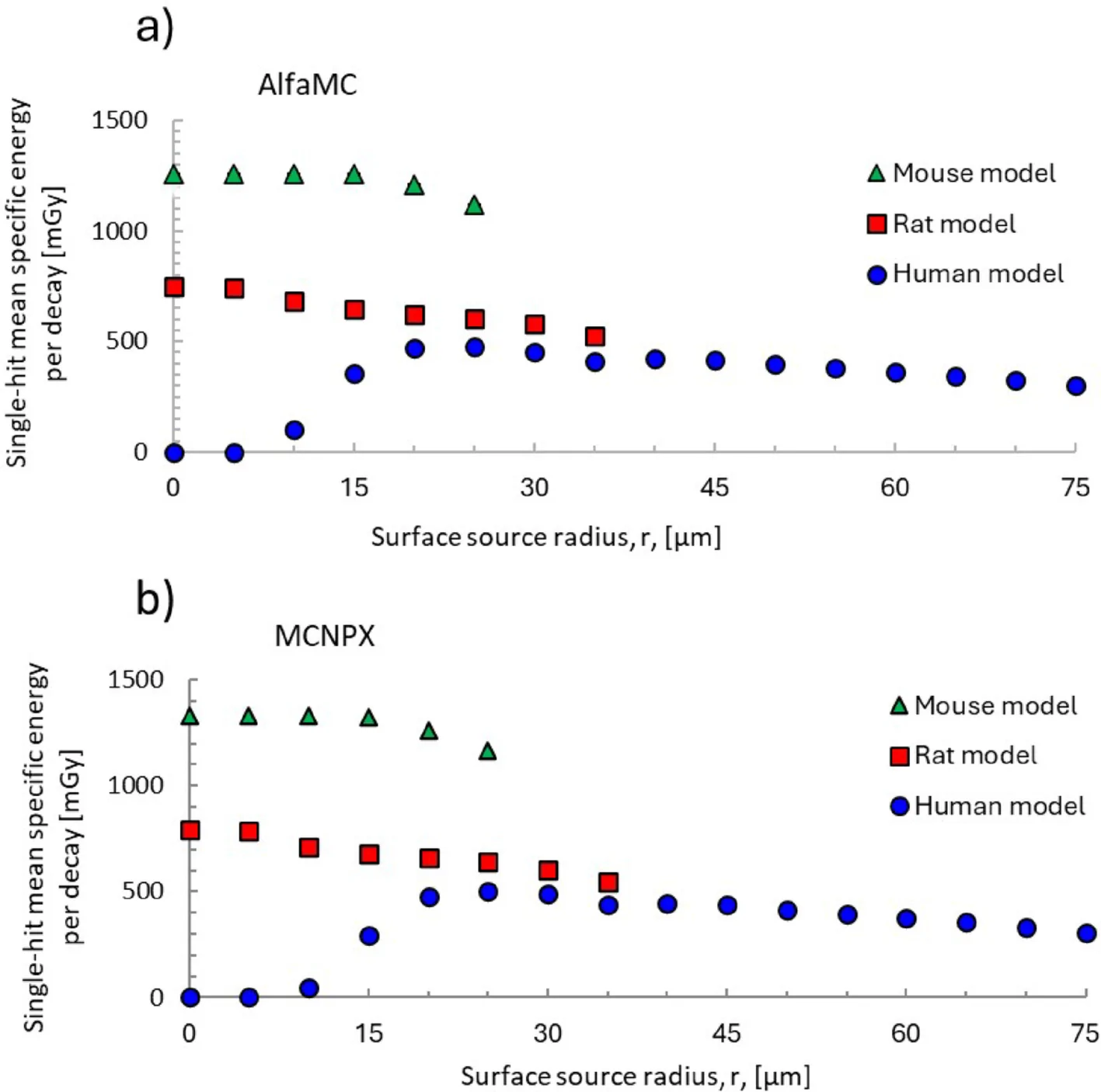
Single-hit mean specific energy per decay simulated with (a) AlfaMC and (b) MCNPX [21, 22] for ^211^At uniformly distributed on the surface of concentric spheres and the six nuclei as the targets

AlfaMC results for ^211^At uniformly distributed on the surface of concentric spheres fixed at the center of the follicle lumen are presented in Fig. 4a. The six follicle cell nuclei were the targets. The single-hit mean specific energy per decay was plotted as the function of the sphere radius which was increased with 5 μm increment until the layer of follicle cells were reached for respective species. Figure 4b shows corresponding results from simulations performed by MCNPX [21, 22]. The ratio between the AlfaMC and the MCNPX data was near one except for the human model; surface-source radius of 10- and 15-mm, where a respective ratio of 2.2 and 1.2 was obtained (not presented). The standard deviation was calculated for each measurement and presented as error bars, but due to their low values they are not visible in Fig. 4.

### Multiple thyroid follicle models

**Fig. 5:**
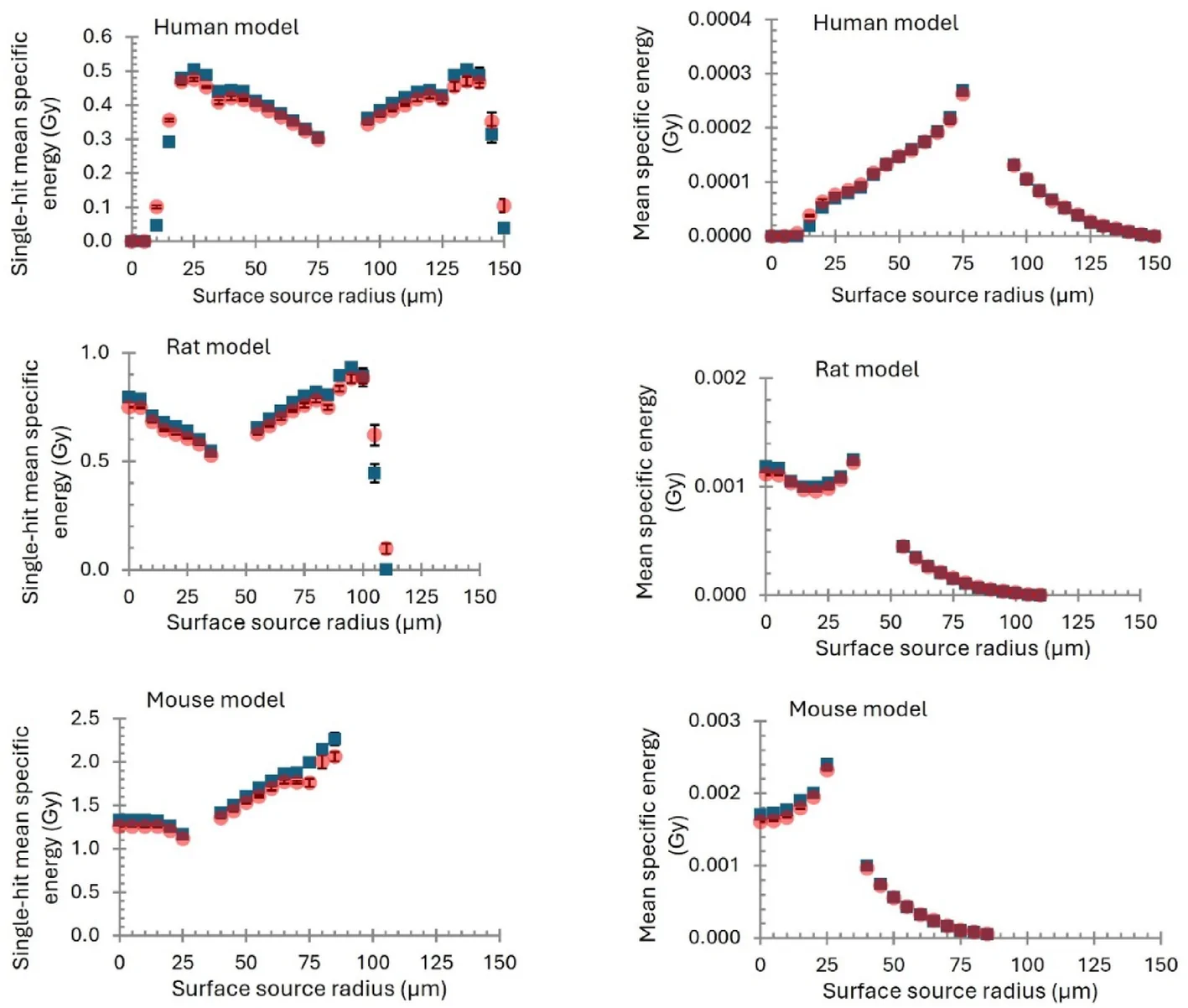
The single-hit mean specific energy (left-column diagrams) and the mean specific energy (right-column diagrams) as function of the surface source radius for the human, rat and mouse models, simulated with AlfaMC (red filled circles) and MCNPX [21, 22] (blue boxes)

Figure 5 shows single-hit mean specific energy and the mean specific energy for ^211^At located in the lumens of the multiple thyroid follicles model an uniformly distributed on the surface of the concentric spheres. The radius of the spheres varied from 0 to 150 μm with 5 μm increments until the basal layer of follicle cells was reached at 75 μm; at 35 and at 25 μm for the human, rat and mouse models, respectively. The increments then continued from the second layer of follicle cells at 95 μm to 150 μm, at 55 μm to 110 μm and at 40 μm to 85 μm, for the human, rat and mouse models, respectively. In all simulations the targets were the six follicle cell nuclei in the central follicle. The standard deviation was calculated for each measurement and is presented as error bars, but due to their low values they are not visible for all data points.

A strong agreement between the data from the two codes was observed for the human, rat and mouse models with constantly higher resemblance for the comparison between the mean specific energy.

## Discussion

The overall purpose of this work was to compare microdosimetric parameters determined by the MC code AlfaMC to MCNPX and Geant4. Mean specific energy and single-hit mean specific energy and single-hit specific energy distribution were investigated. The mean specific energy represented the average specific energy per mass including zero-hit events. It can be used for S-values calculation of the mean absorbed doses. The single-hit mean specific energy contained information about the average specific energy deposited exclusively by single events. The mean absorbed dose can be calculated for a certain number of alpha particles hitting the target by using the average value per hit or by using the probability distribution and a random number generator for sampling of the contribution of each alpha particle.

The study was applied to ^211^At exposure in thyroid follicle models for man, rat and mouse. The models that we used were all multicellular, either simulating a single thyroid follicle or multiple thyroid follicles. The multifollicle models reflected the situation in the entire thyroid gland and were the most interesting models that best resembled in vivo or clinical situation.

However, we also found of interest to present the dosimetric contribution from different source distributions within a follicle to better understand the differences obtained for the different species. That might help the reader to better interpret the data and use these data when optimizing the absorbed dose to the thyroid to avoid side effects from treatment with ^211^At-labelled pharmaceuticals. Several papers reported recently experimental and/or microdosimetric studies on cells in vitro with similar aims, for example [27, 28, 29]. Furthermore, several dosimetric studies on different radionuclide distributions for other radionuclides were done for single thyroid follicle models, for example [30, 31, 32]. Therefore, we decided to present results also for the single follicle model to facilitate comparison between studies and radionuclides. Astatine is a halogen that can occur in many valence states, both positive and negative. The knowledge of the physiological behavior of astatine is very limited in man and limited in animals. Sometimes, astatine is thought to behave analogously to halogen iodine, and there are some similarities, but also major differences demonstrated in animals. There were major differences in biodistribution in various organs and tissues in the body, studied both in mice and rats [10, 11]. One similarity was that astatine was also transported into the thyroid follicle cell and then through the cell and into the follicle lumen according to studies in an in vitro-reconstituted thyroid epithelium of porcine thyrocytes cultured in bicameral chambers [24]. However, the amount of astatine transported was lower than that for iodide, and the results demonstrated that astatine was not only transported via NIS (sodium-iodide transporter) as iodide is, but also via other transport mechanisms not related to NIS [24]. Iodide was also known to be organified to thyroglobulin before entering the lumen, while no or very little organification was assumed for astatine.

Thus, the four ^211^At distributions considered here simulated the potential localization of free ^211^At in the follicle cells and in the follicle lumen, distributions that are likely, although localization within the cell nuclei is less probable. Furthermore, ^211^At bound to a pharmaceutical could be simulated by the distribution on the surface of the concentric spheres representing the basolateral membrane of the follicle cells.

The mean specific energy,$\left\langle z \right\rangle$and the single-hit mean specific energy,$\left\langle {z_{1} } \right\rangle$, were determined for uniform and non-uniform ^211^At-distributions within the single thyroid model for mouse, rat and man using AlfaMC. Difference between 1.7 and 4.6%, was observed for the uniform distribution of ^211^At between the results obtained by AlfaMC and the MCNPX data published earlier [21, 22]. The AlfaMC values of $\left\langle z \right\rangle$ and $\left\langle {z_{1} } \right\rangle$ were lower in comparison with MCNPX for each species (Table 1). AlfaMC results showed better agreement with the results obtained by Geant4, percentage difference up to 1.7%. Also, in this comparison, AlfaMC returned in general lower $\left\langle z \right\rangle$ and $\left\langle {z_{1} } \right\rangle$ values. The single-hit mean specific energy distribution, f(z_1_), for respective species and source location simulated with AlfaMC, MCNPX and Geant4 showed good correlation. The maximum and the minimum values of the probability density functions occurred at approximately the same specific energy for respective species and source location.

The ratio between $\left\langle {z_{1} } \right\rangle$ simulated with AlfaMC and MCNPX for respective species, in the cases where ^211^At was uniformly distributed on concentric spheres (to represent a heterogeneous distribution in the follicle lumens), varied from 0.93 to 1.0 for most of the data points. Also, in this case AlfaMC underestimated the$\left\langle {z_{1} } \right\rangle$ compared to MCNPX. However, for the human model two data points differed with 20% and 120%, respectively. The distance between the source location and the center of the targets were 70 and 65 μm for respectively data point, which is approximately the maximum range of the emitted 7.45 MeV alpha-particles. AlfaMC results from the non-uniform distribution showed good resemblances with MCNPX [21, 22] for both $\left\langle z \right\rangle$ and$\left\langle {z_{1} } \right\rangle$. Similarities in the shape of the curves were observed for respective species (Fig. 5).

Altogether, the differences between the results from AlfaMC, MCNPX and Geant4 were partly due to their different ways of describing the transport and the energy deposition of the alpha-particles. The ASTAR database, on which AlfaMC was based, is part of NIST standard reference database including stopping-power and range tables for various particles calculated according to methods described in ICRU Report 49 [33]. ASTAR has stated the uncertainties in collision stopping power for high energies (> 2 MeV) of the alpha-particle to be 1–4%. For energies below 100 keV, which is in the energy region of the Bragg-peak-tail, the uncertainties exceeded 10% [26]. Hence small difference in the Bragg-peak-tail may lead to fluctuations in the energy deposition.

MCNPX and Geant4 employed physics models using tabulated or analytical cross sections. Approximations and uncertainties in the interaction modelling may cause differences in the evaluation of microdosimetric quantities. MCNPX was based on a condensed history approach where many collision processes were grouped together in a single step and stopping power data from protons scaled to alpha-particles were used.

The stopping power computed by Geant4 was benchmarked against the same NIST/ASTAR stopping-power database applied in AlfaMC. The transport of secondary electrons was controlled by the production cutoff and step limitation set to 1 micrometer. Secondary electrons with a range below the cutoff limitation deposited their energy locally.

Production cuts were not used by Geant4 DNA processes since all interactions were explicitly simulated. The activation of Gean4 DNA Physics only in the nucleus target did not substantially alter Geant4 results. Although the difference against MCNPX decreased it was still within statistical uncertainty. Long calculation time prohibited the implementation of full Geant4 DNA physics model, using DNA Physics processes in the entire geometry World. Simulation time of 1000 particles of about 1 h was observed, with at least 5.10^7^ particles needed to obtain statistical uncertainty below 1%.

The usage of DNA Physics simulating event-by-event interactions of alpha particles is commonly recognized for track evaluation and energy deposition at nanometer scale. There are still open questions at micrometer scale. The effect of combining regions with different physics models to achieve acceptable computation times needs further investigation. In particular, the effect of extending the DNA Physics region beyond the target could be addressed in future work.

The simulation geometry was another factor that could possibly influence the result. The voxel size of the phantom was found to affect microdosimetric results of MCNPX and Geant4 [34]. However, the systematic difference obtained here between results for man, rat and mouse suggested negligible size effect.

Intercomparison of microdosimetric parameters obtained by different models and MC codes simulating alpha-particle transport was used as an effective way for validation of a microdosimetry model. Agreement within 5% with published results was considered as good [35]. Deviations up to 9% between analytical model and Geant4-DNA results for spherical geometries with diameter of 1 to 10 μm was acceptable [26]. Dosimetry study at sub-cellular scale for the Auger-electron emitter (^99m^Tc) using Monte Carlo N-particle (MCNP6) code showed an agreement within 3% compared to the MCNPX results using the thyroid follicle model [21, 22, 37].

The importance of information provided by microdosimetry in terms of specific-energy distributions for therapeutic applications and the understanding of the cellular response to alpha-particles was outlined in [38]. The disadvantage of microdosimetry related to intensive computational issues tends to decrease opening possibilities of advanced dosimetry systems for targeted alpha therapy. Particle transport at macroscopic level was coupled to microdosimetric kinetic model for assessment of patient specific dose delivery and biological effects [39].

Microdosimetric estimations of the exposure of thyroid are important when ^211^At-labeled pharmaceuticals are used for treatment of non-thyroid cancers. The thyroid is beside bone marrow and potentially the kidneys the risk organs for this treatment modality. The reason for focusing on thyroid is that “free ^211^At” is released and to high extent accumulates in the thyroid partly via NIS. Clinical trials are most often preceded by preclinical studies on mice and rats. As shown in the present study, the size differences in thyroid follicle lumen, cell thickness and diameter of the cell nucleus clearly show that translation of absorbed doses to thyroid from rodents to man will differ substantially, especially when it comes to the contribution from ^211^At in the follicle lumen, due to the limited range of the emitted alpha particles. For a more realistic dosimetric estimation in man, it is important with realistic follicle model(s) that includes the larger follicle size in man, since the absorbed dose to follicle cells and their nuclei will be lower than if ^211^At is assumed to be homogeneously distributed in the thyroid gland. Still, for radiation safety of the patient, the uptake and retention of ^211^At in the thyroid should be reduced, which to a high extent can be made by single or repeated administrations of “thyroid blocker” like stable iodide or perchlorate [40]. When evaluating different geometric and biokinetic/functional thyroid models for further thyroid protection strategies, fast and common user friendly MC simulations are preferable. The data presented in the present study show that AlfaMC would be preferable compared to MCNPX and Geant4 for such studies.

## Conclusion

The dosimetric results from AlfaMC agreed within 5% to those from MCNPX and Geant4, which demonstrates the accuracy of the AlfaMC code for microdosimetric calculations at the cellular and subcellular level of the thyroid. Due to the much shorter calculation times of AlfaMC compared to MCNPX and Geant4 and its specific customization to alpha particle transport, AlfaMC could preferably be used when comparing microdosimetric parameters between species or when evaluating different strategies to reduce the absorbed dose to thyroid when treating patients with ^211^At-labelled pharmaceuticals.

## Data Availability

data generated or analyzed during this study are included in this published article.
